# Supplementary material for: Highly replicating hepatitis C virus variants emerge in immunosuppressed patients causing severe disease
Source: Nat Commun. 2025 Dec 7;16:10948. doi: 10.1038/s41467-025-67174-w (PMC12686413; doi:10.1038/s41467-025-67174-w)
Supplement: Supplementary file 1 — Supplementary Information [file 41467_2025_67174_MOESM1_ESM.pdf]

Highly replicating hepatitis C virus variants emerge in immunosuppressed patients causing severe disease

Paul Rothhaar, Tomke Arand, Ha Gyu-Thomas Seong, Christian Heuss, Margaret Tulessin, Zhiqing Wang, Colin Förster, Alina C. Schneider, Jocelyn Quistrebert, Haiting Chai, Marvin Reineke, Louise Benning, Jonathan Honegger, Maike Hofmann, Robert Thimme, Jörg Timm, STOPHCV investigators, Paul Schnitzler, Uta Merle, Naglaa H. Shoukry, Julie Bruneau, Chaturaka Rodrigo, Andrew Lloyd, Rowena A. Bull, M. Azim Ansari, Carolin Mogler, John McLauchlan, Xavier Forns, Sofía Pérez-del-Pulgar, Volker Lohmann

Supplementary Figure S1-S12

Supplementary Table S1-S3

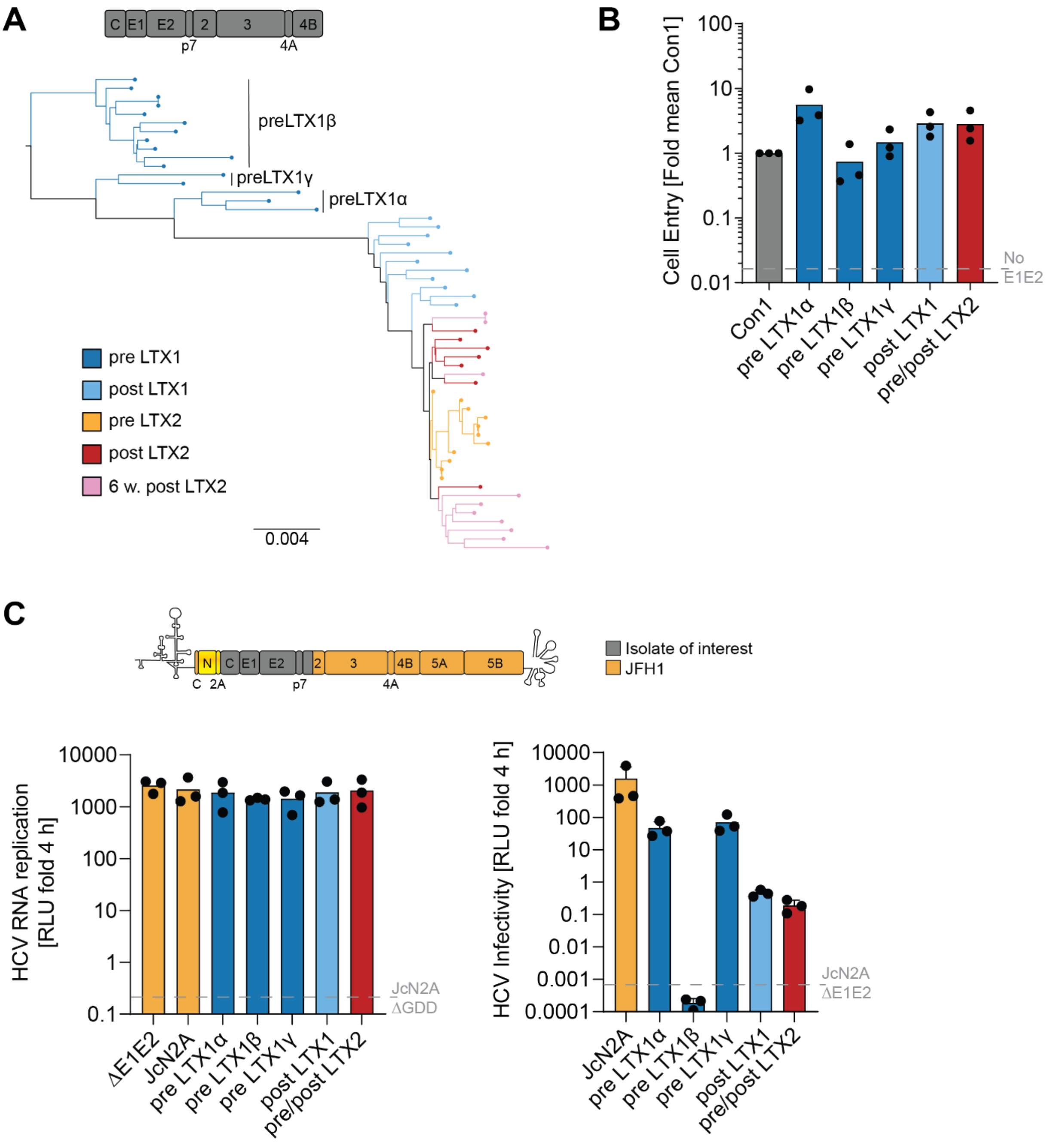

Fig. S1: Phenotypic characterization of the evolution of the assembly module in the GLT1 patient. A) Phylogenetic tree for the first PCR fragment which includes the whole assembly module. B) HCVpp bearing HCV envelope proteins of the indicated isolates on their surface were added to Huh7-Lunet CD81 cells. After 72 h, cells were lysed, and infectivity was determined based on a luciferase reporter in the HCVpp. Measurements were first normalised to the input determined via SG-PERT and then normalised to the Con1 reference isolate. Particles without envelope proteins (No E1E2) served as negative control. C) Huh7-Lunet CD81 cells were transfected with the chimeric full-length HCV reporter construct depicted in the upper panel. It combines the sequence of the isolate of interest up to the C3 junction site in NS2 with the remaining part of the JFH1 isolate[1]. RNA replication was determined at 72 hours post transfection (left panel) and the supernatant was used to infect naïve Huh7-Lunet CD81 cells. At 72 hours post infection, cells were lysed, and infectivity was determined via luciferase measurement (right panel). A replication deficient JcN2A variant (JcN2A ΔGDD) and a JcN2A variant lacking envelope proteins (JcN2A ΔE1E2) were used as negative controls for replication and infection respectively.

B&C) Data are from three independent biological replicates measured in technical duplicates. Each dot depicts the result of one replicate and the bar indicates the mean of all replicates.

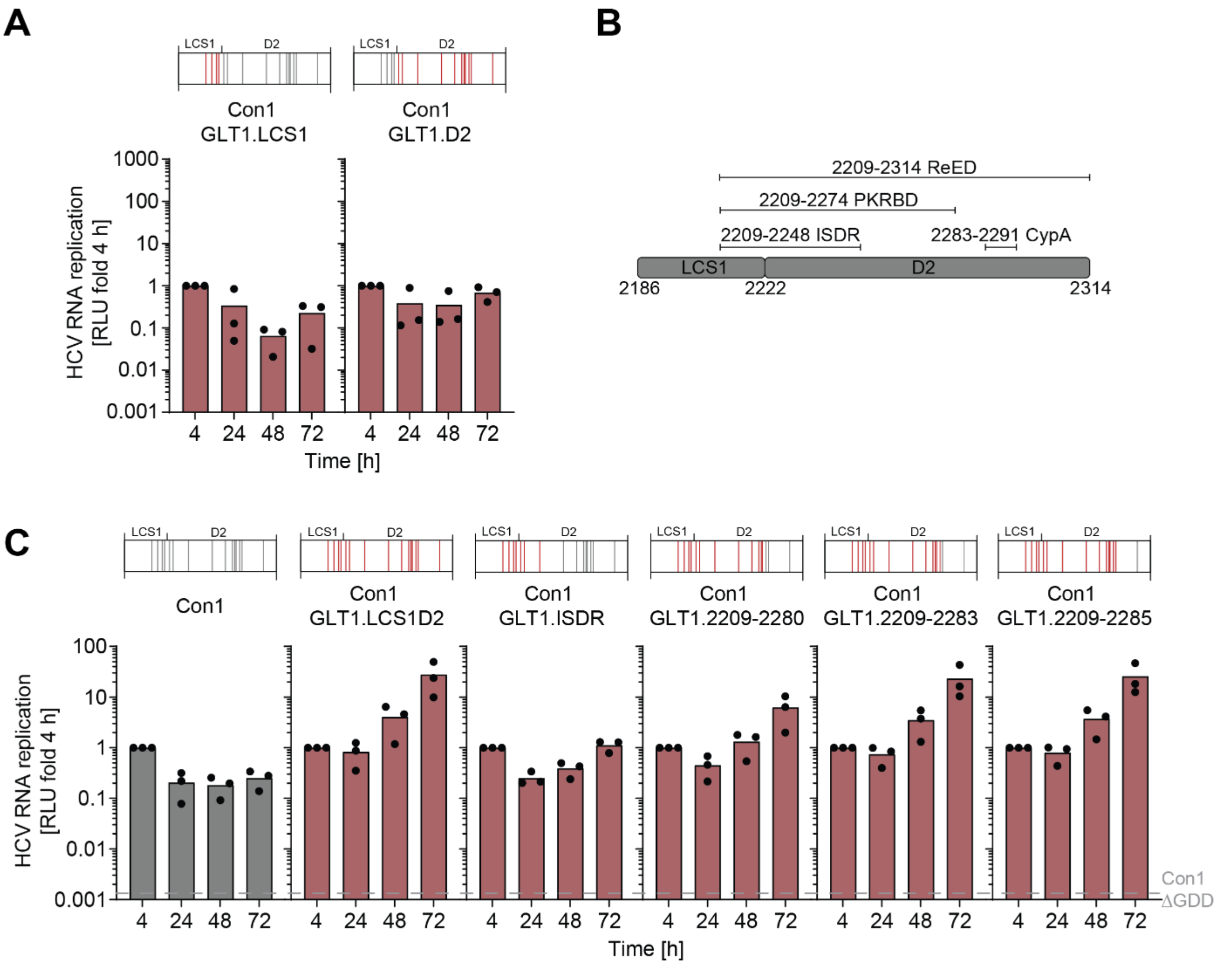

Fig. S2: Mapping of relevant residues in the ReED. A&C) SGRs of the indicated constructs were electroporated into Huh7-Lunet SEC14L2 cells, luciferase activity in cell lysates (RLU) was quantified as a correlate of RNA replication efficiency at the given time points and normalised to 4 h to account for differences in transfection efficiency. Con1 ΔGDD served as a replication deficient negative control. Data are from three independent biological replicates measured in technical duplicates. Each dot depicts the result of one replicate and the bar indicates the mean of all replicates. B) Schematic illustrating LCS1D2, informing about the location of the ReED, the ISDR[2], the PKR binding domain (PKRBD)[3] and the Cyclophilin A (CypA) binding site[4].

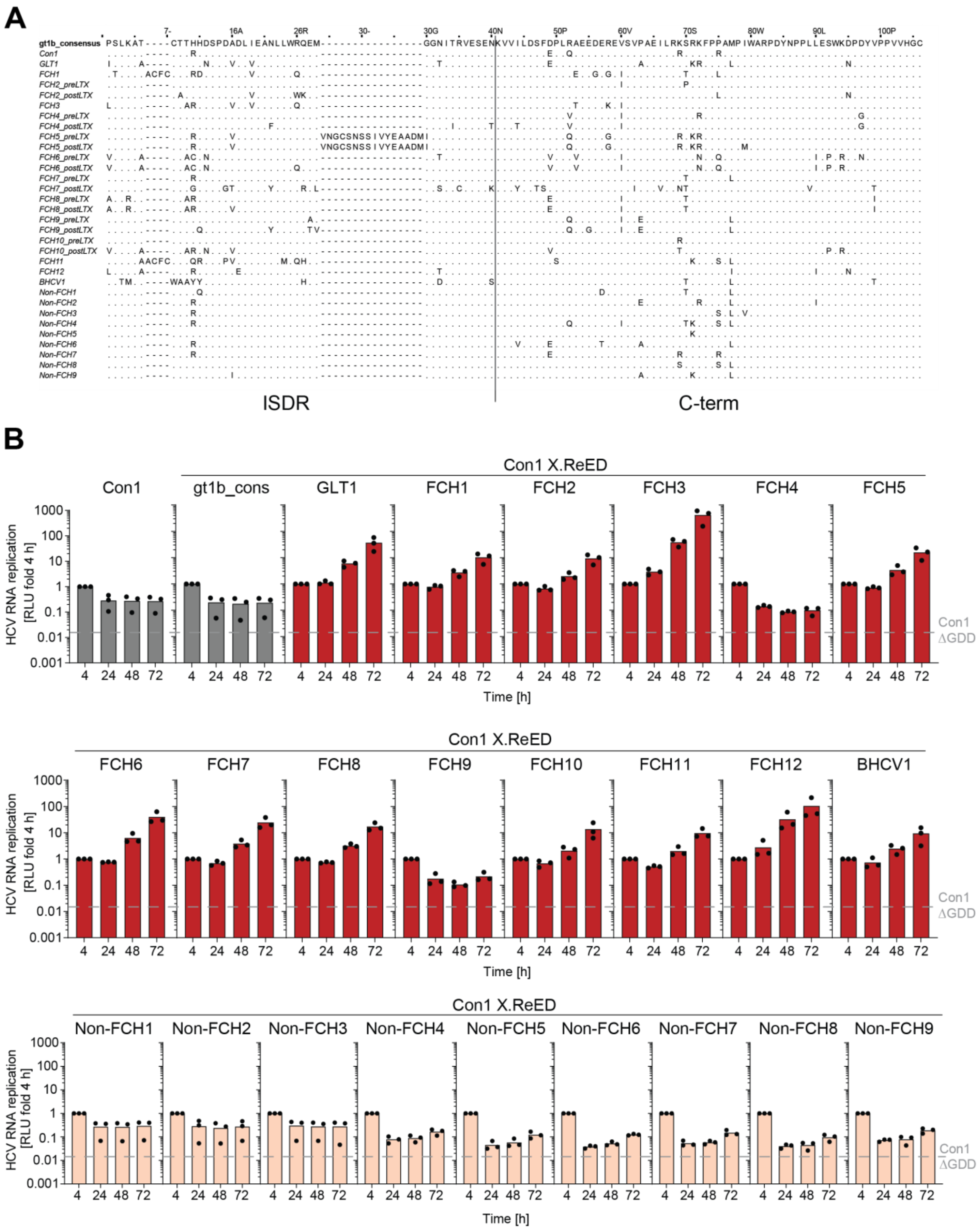

Fig. S3: Replication kinetics of SGRs from the FCH cohort. A) Amino acid alignment of all ReED sequences from the FCH cohort, using the genotype 1b consensus ReED as a reference. Dots indicate an amino acid being identical to the gt1b consensus. Note that also pre LTX sequences are shown when available. B) SGRs of the indicated constructs were electroporated into Huh7-Lunet SEC14L2 cells, luciferase activity in cell lysates (RLU) was quantified as a correlate of RNA replication efficiency at the given time points and normalised to 4 h to account for differences in transfection efficiency. Con1 ΔGDD served as a replication deficient negative control. Data are

from three independent biological replicates measured in technical duplicates. Each dot depicts the result of one replicate and the bar indicates the mean of all replicates.

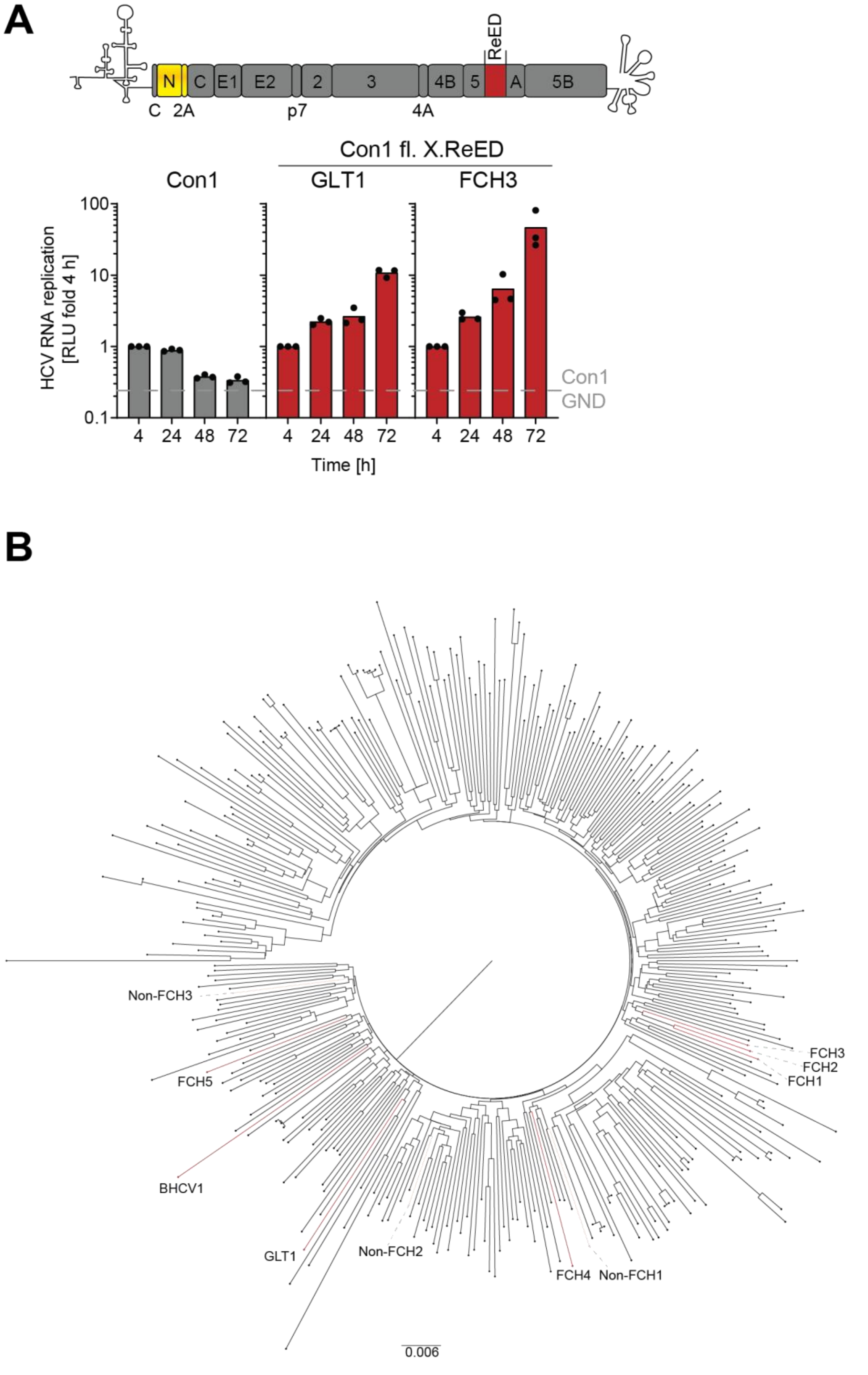

Fig. S4: Full-length and phylogenetic studies on the FCH cohort. A) Full-length replicons of the indicated constructs were electroporated into Huh7-Lunet SEC14L2 cells, luciferase activity in cell lysates (RLU) was quantified as a correlate of RNA replication efficiency at the given time points and normalised to 4 h to account for differences in transfection efficiency. Con1 GND

served as replication deficient negative control. Data are from three independent biological replicates measured in technical duplicates. Each dot depicts the result of one replicate and the bar indicates the mean of all replicates. B) Phylogenetic tree of gt1b isolates retrieved from ViPR[5]. Highlighted are the 7 FCH (red) and 3 non-FCH (apricot) patients for which complete replicase sequences post LTX were available. Accession numbers: FCH1 - MK092096, FCH2 - MK092098, FCH3 - MK092100, FCH4 - MK092102, FCH5 - MK092104, BHCV1 - HQ719473, GLT1 - OM222702, Non-FCH1 - MK092106, Non-FCH2 - MK092108, Non-FCH3 - MK092110.

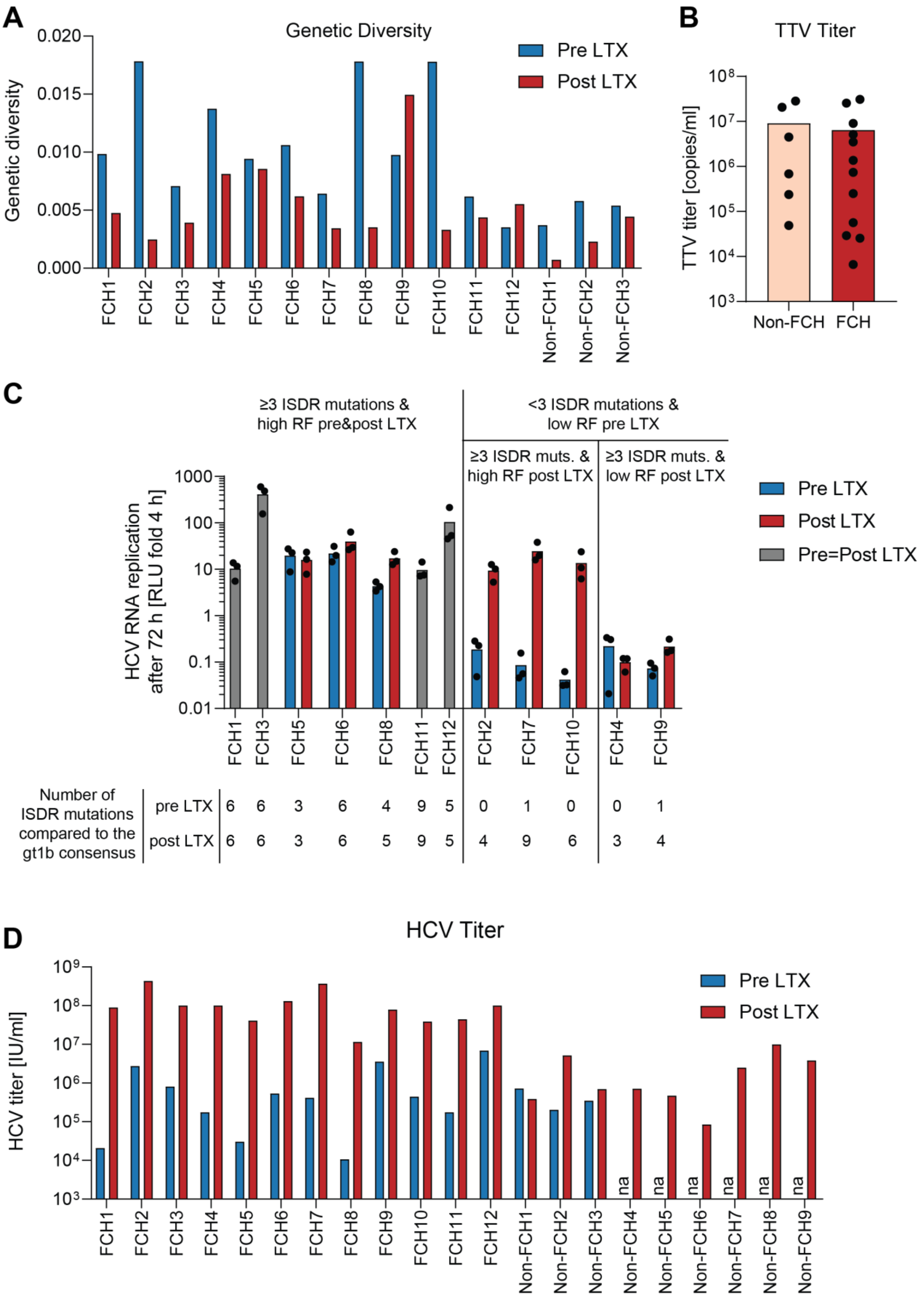

Fig. S5: Patient characteristics and evolution of RF in the FCH cohort. A&D) Data on genetic diversity based on NGS analysis of NS5B (A) and HCV serum titers pre and post LTX for FCH1-12 and non-FCH1-3 (D) were previously published in bulk[6] and are here stratified by patient. No genetic diversity data and no pre LTX titers were available for non-FCH4-9. B) Torque teno virus

(TTV) titers in post LTX patient serum were determined via qPCR. C) For the determination of ISDR mutations (lower panel), an insertion was counted as one mutation irrespective of its length. SGRs of the indicated constructs were electroporated into Huh7-Lunet SEC14L2 cells, luciferase activity in cell lysates (RLU) was quantified as a correlate of RNA replication efficiency and normalised to 4 h to account for differences in transfection efficiency. Displayed is the time point 72 h post electroporation, data for the post LTX samples is taken from Fig. S3B. Data are from three independent biological replicates measured in technical duplicates. Each dot depicts the result of one replicate and the bar indicates the mean of all replicates.

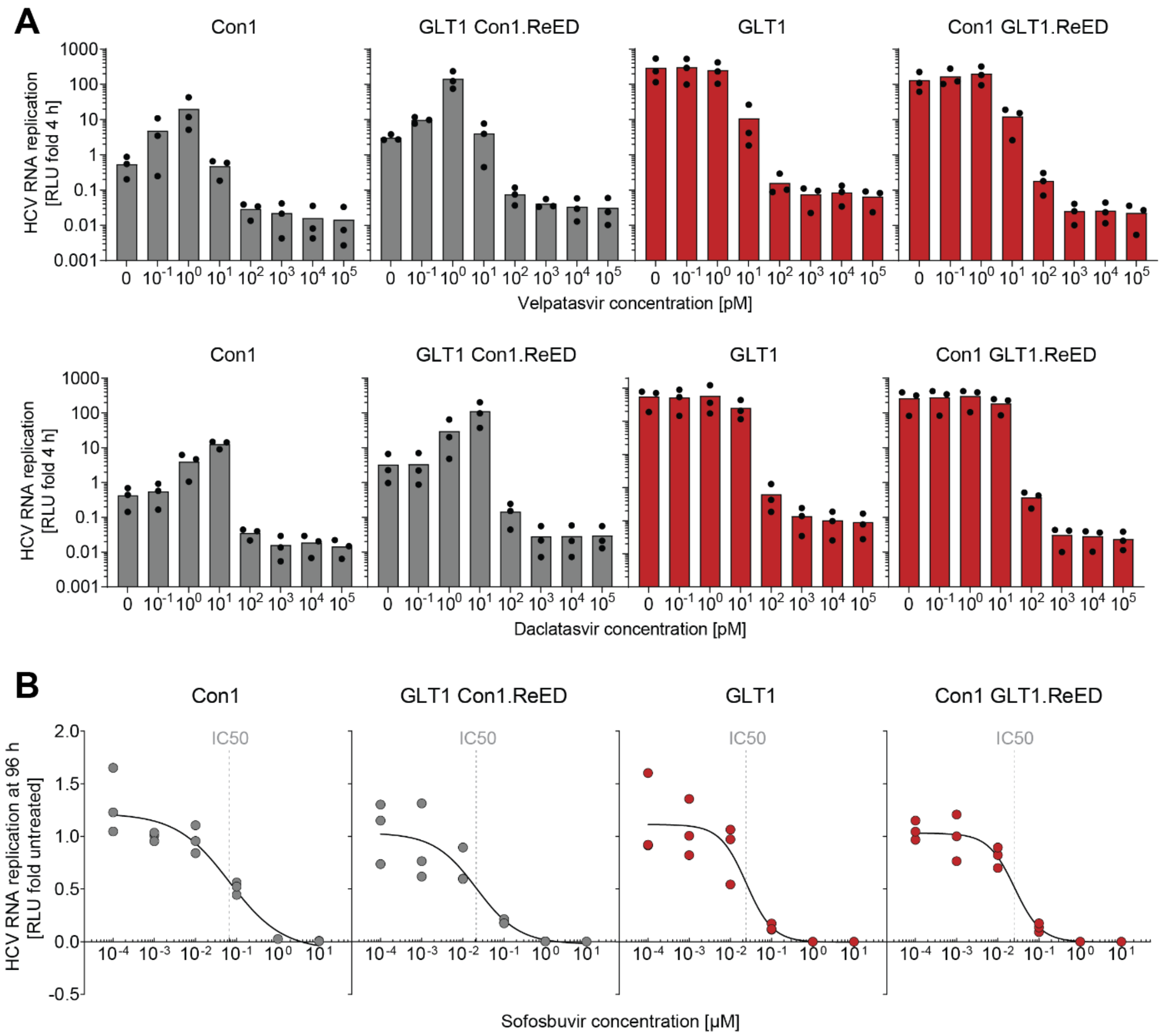

Isolate	Con1	GLT1	Con1 GLT1.ReED	GLT1 Con1.ReED
IC50	0.06623	0.02436	0.02542	0.02142

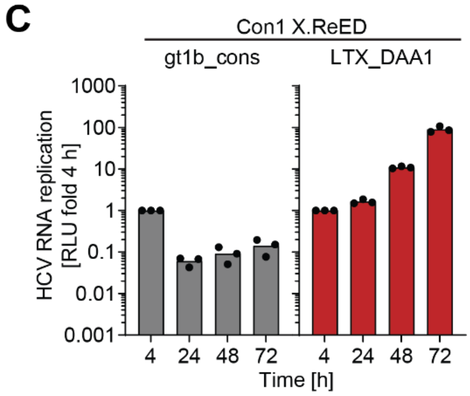

Fig. S6: DAA treatment of SGRs with different RF. SGRs of the indicated constructs were electroporated into Huh7-Lunet SEC14L2 cells, luciferase activity in cell lysates (RLU) was quantified as a correlate of RNA replication efficiency at the given time points and normalised to 4 h to account for differences in transfection efficiency (A&C) or normalised to an untreated control (B). A&B) Replication 96 h after electroporation is depicted, treatment with the indicated drug and concentration was performed 24 h after electroporation. IC50 of Sofosbuvir treatment is depicted as a dashed grey line (B, upper panel) and also shown as a numeric value (B, lower

panel). Data are from three independent biological replicates measured in technical duplicates. Each dot depicts the result of one replicate, and the bar indicates the mean of all replicates (A&C), or the black line indicates the non-linear fit used for IC50 determination (B).

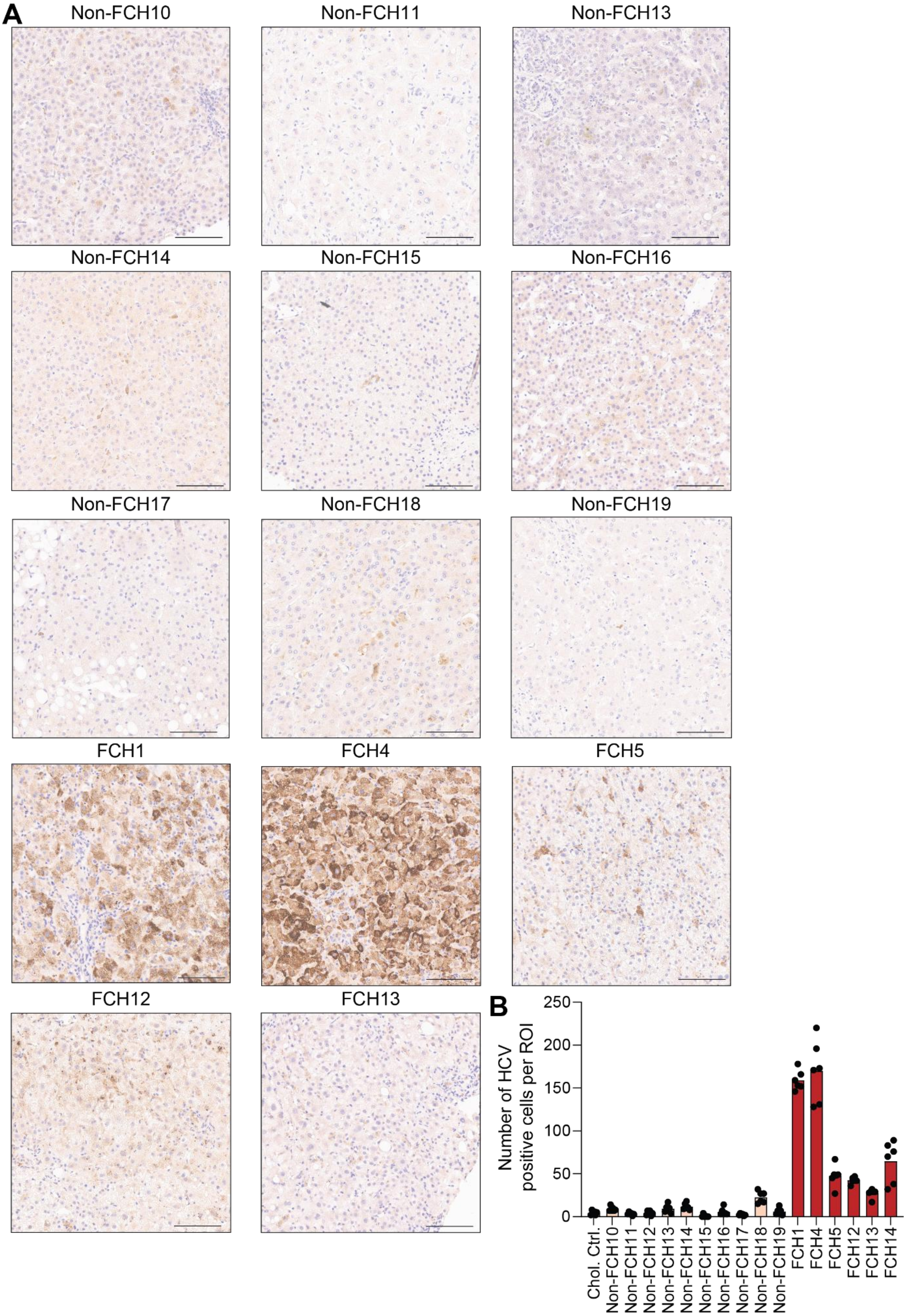

Fig. S7: Representative images of IHC stained liver tissue sections. A) An HCV NS5A specific antibody was used for staining. Representative images showing one quantified region of interest (ROI) per patient for the patients not displayed in Fig. 3E. Scale bars indicate 100 µm. B) Quantification displayed in Fig. 3E stratified by patient. Chol. Ctrl. represents an HCV negative cholestatic liver. Note that only patients FCH1, 4, 5 and 12 overlap with the patients characterised in Fig. 3A-C due to limited availability of samples.

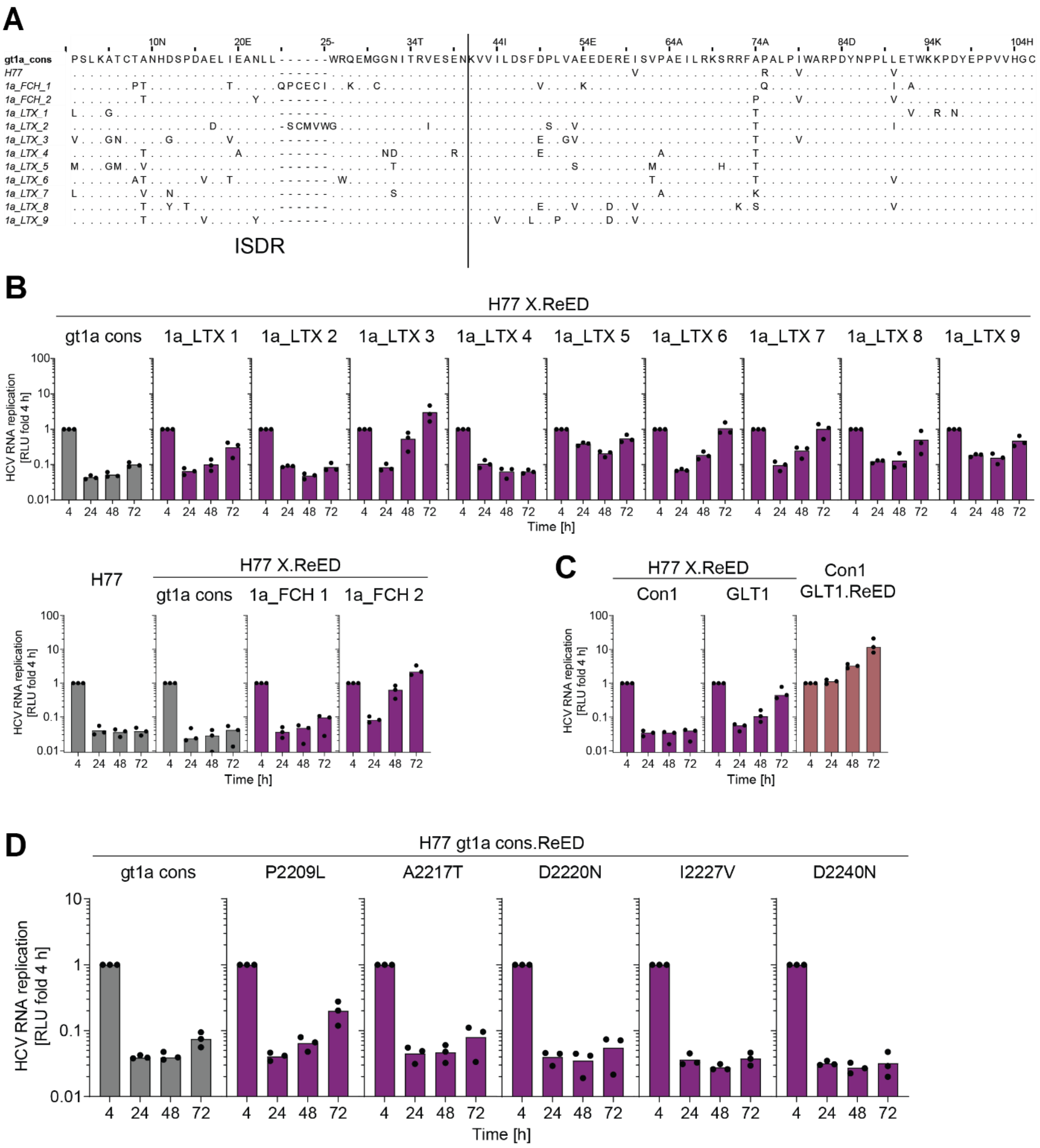

Fig. S8: Replication kinetics of chimeric SGRs from gt1a. A) Amino acid sequence alignment of patient derived ReEDs compared to the gt1a consensus ReED. Dots indicate an amino acid being identical to the gt1a consensus. B-D) SGRs of the indicated constructs were electroporated into Huh7-Lunet SEC14L2 cells, luciferase activity in cell lysates (RLU) was quantified as a correlate

of RNA replication efficiency at the given time points and normalised to 4 h to account for differences in transfection efficiency. Data are from three independent biological replicates measured in technical duplicates. Each dot depicts the result of one replicate and the bar indicates the mean of all replicates.

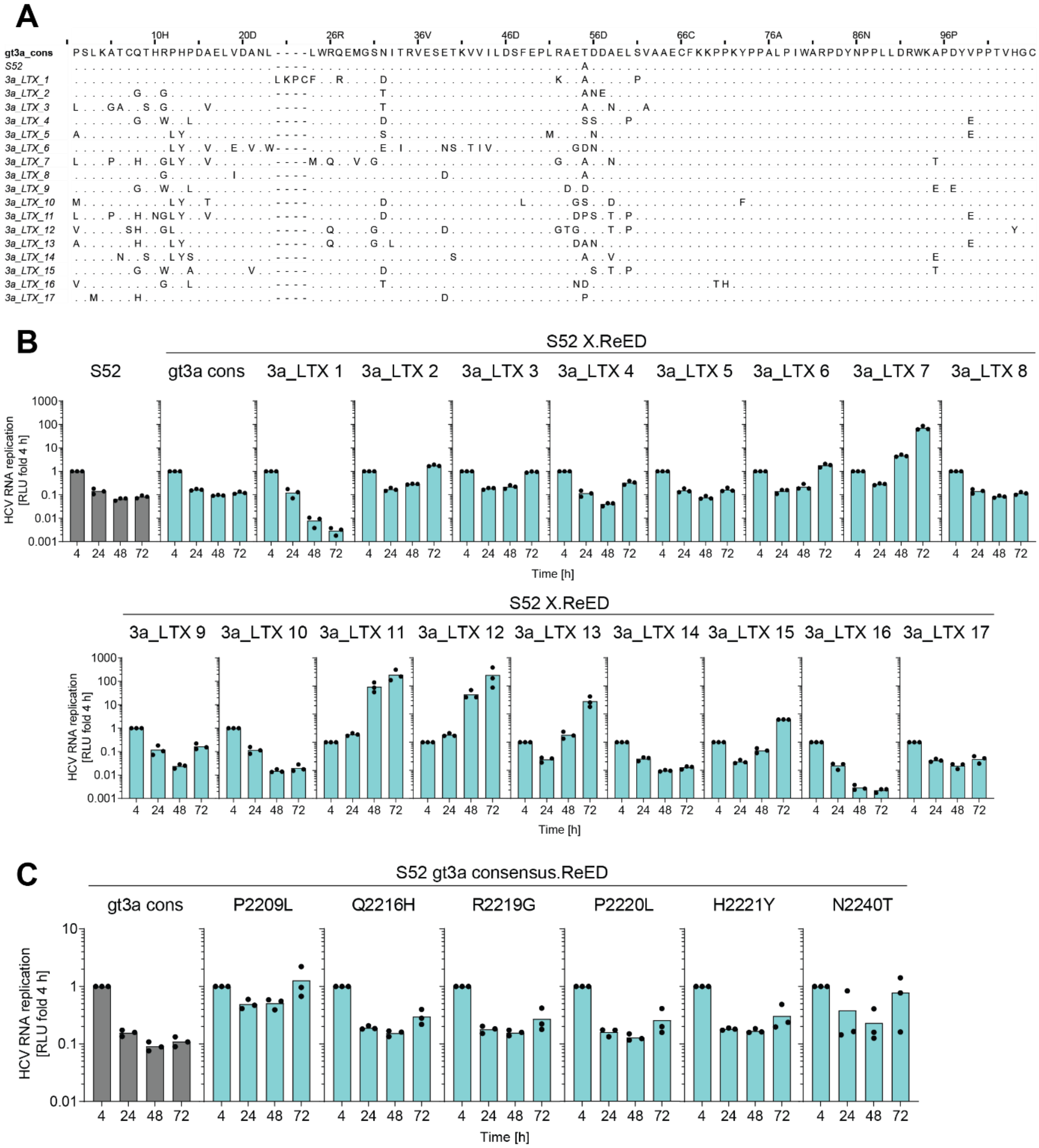

Fig. S9: Replication kinetics of chimeric SGRs from gt3a. A) Amino acid sequence alignment of patient derived ReEDs compared to the gt3a consensus ReED. Dots indicate an amino acid being identical to the gt3a consensus. B&C) SGRs of the indicated constructs were electroporated into Huh7-Lunet SEC14L2 cells, luciferase activity in cell lysates (RLU) was quantified as a correlate of RNA replication efficiency at the given time points and normalised to 4 h to account for differences in transfection efficiency. Data are from three independent biological replicates

measured in technical duplicates. Each dot depicts the result of one replicate and the bar indicates the mean of all replicates.

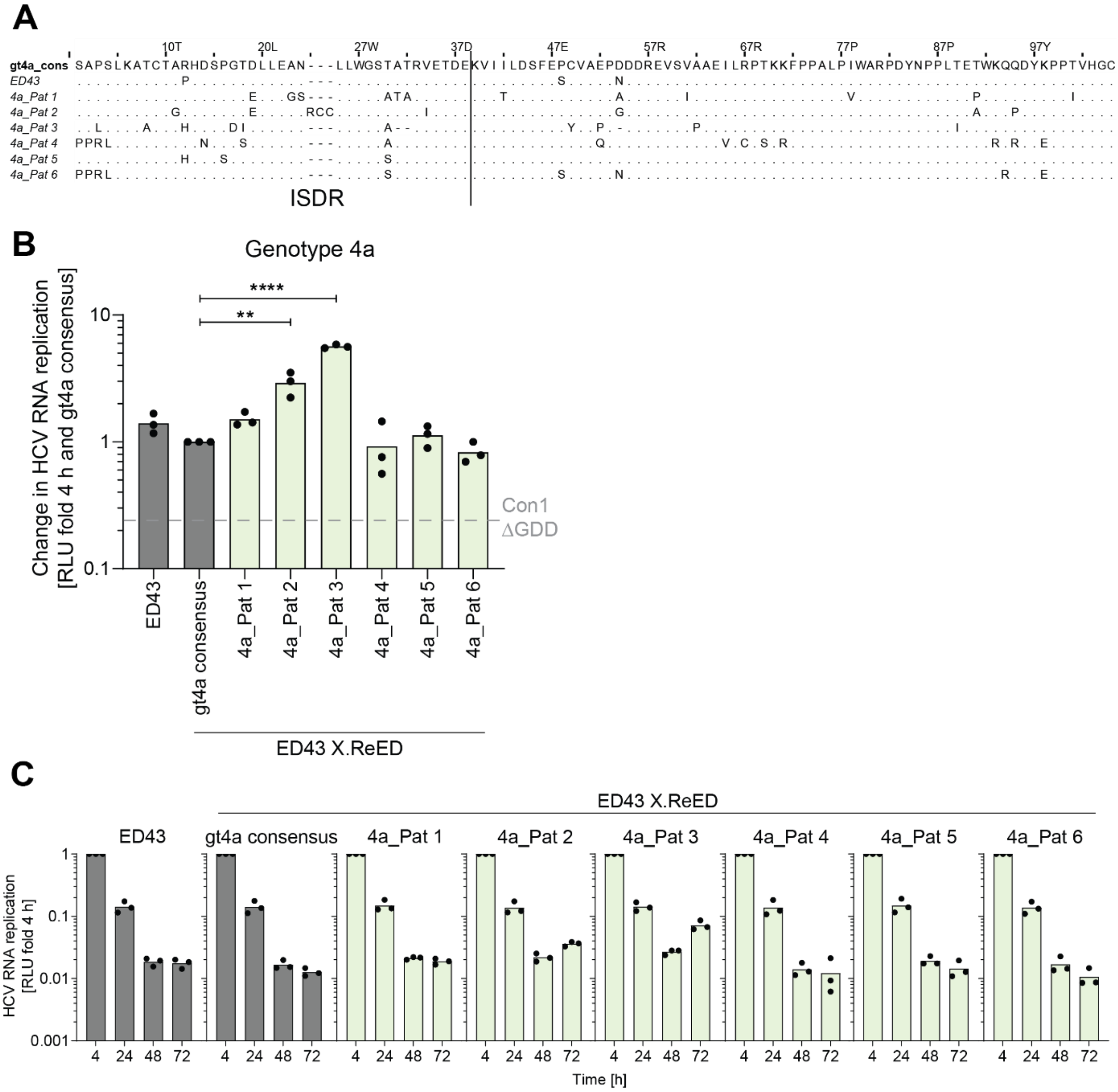

Fig. S10: Replication of chimeric SGRs from gt4a. A) Amino acid sequence alignment of patient derived ReEDs compared to the gt4a consensus ReED. Dots indicate an amino acid being identical to the gt4a consensus. B&C) SGRs of the indicated constructs were electroporated into Huh7-Lunet SEC14L2 cells, luciferase activity in cell lysates (RLU) was quantified as a correlate of RNA replication efficiency at the given time points and normalised to 4 h to account for differences in transfection efficiency (C) and subsequently normalised to the values for the gt4a consensus of the respective replicate (B). Data are from three independent biological replicates measured in technical duplicates. Each dot depicts the result of one replicate, and the bar indicates the mean of all replicates.

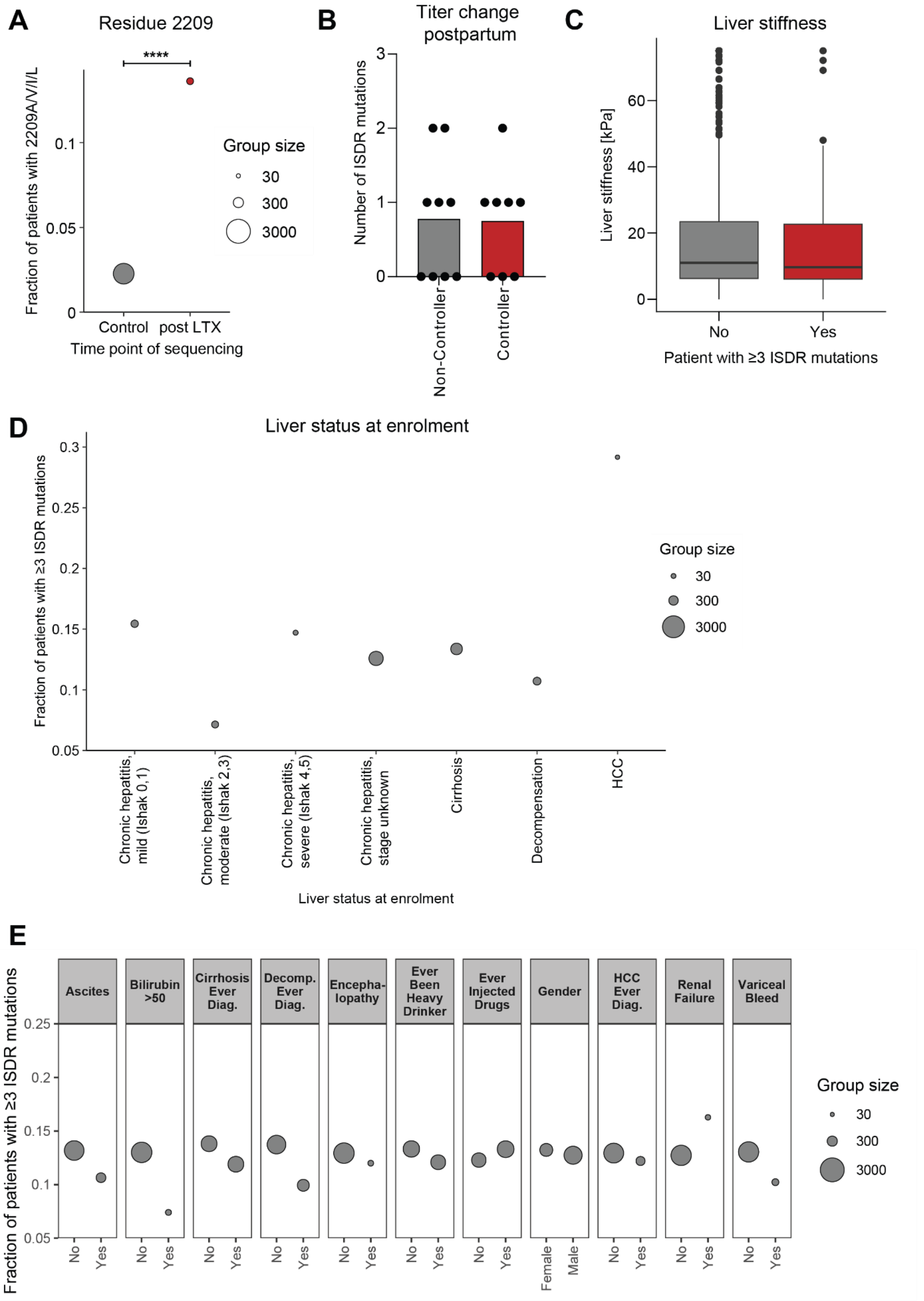

Fig. S11: Sequence signatures of high RF in various clinical contexts. A) Fraction of patients in the HCV Research UK cohort with 2209A/V/I/L stratified whether HCV was sequenced post LTX or not. Statistical significance was determined using a Fisher's exact test. **** $p < 0.0001$. B) HCV

serum titers for each patient were determined in the third trimester of pregnancy and 3 months postpartum. If the HCV titers dropped by more than 1 log postpartum, patients were considered controllers[7]. Combined data of 10 gt1a, 1 gt1b, 1 gt2a, 1 gt2b and 4 gt3a patients. For each patient the number of ISDR amino acid differences compared to the gt specific consensus was determined. No dominant amino acid changes in the ISDR between the two time points was detected in any of the patients thus the results represent both the third trimester of pregnancy and 3 months postpartum time points. C-E) Data based on the HCV Research UK cohort[8]. Data for gts 1a, 1b and 3a were pooled. For each patient's ISDR sequence, the number of amino acid differences compared to the gt specific consensus sequence was determined. If an ISDR had 3 or more mutations, the patient was considered to be infected with a potential high replicator. Post LTX patients were excluded from the analysis. C) Fraction of high replicators in the context of liver stiffness determined via Fibroscan. 112 potential high replicators were compared to 768 potential low replicators. D&E) Fraction of high replicators in the context of liver status at the time point of sequencing (D) or a variety of other contexts (E). Statistical significance was determined using a two-sided Student's t-test (C) or a Fisher's exact test (D&E). In none of the cases, a significant enrichment of potential high replicators was detected.

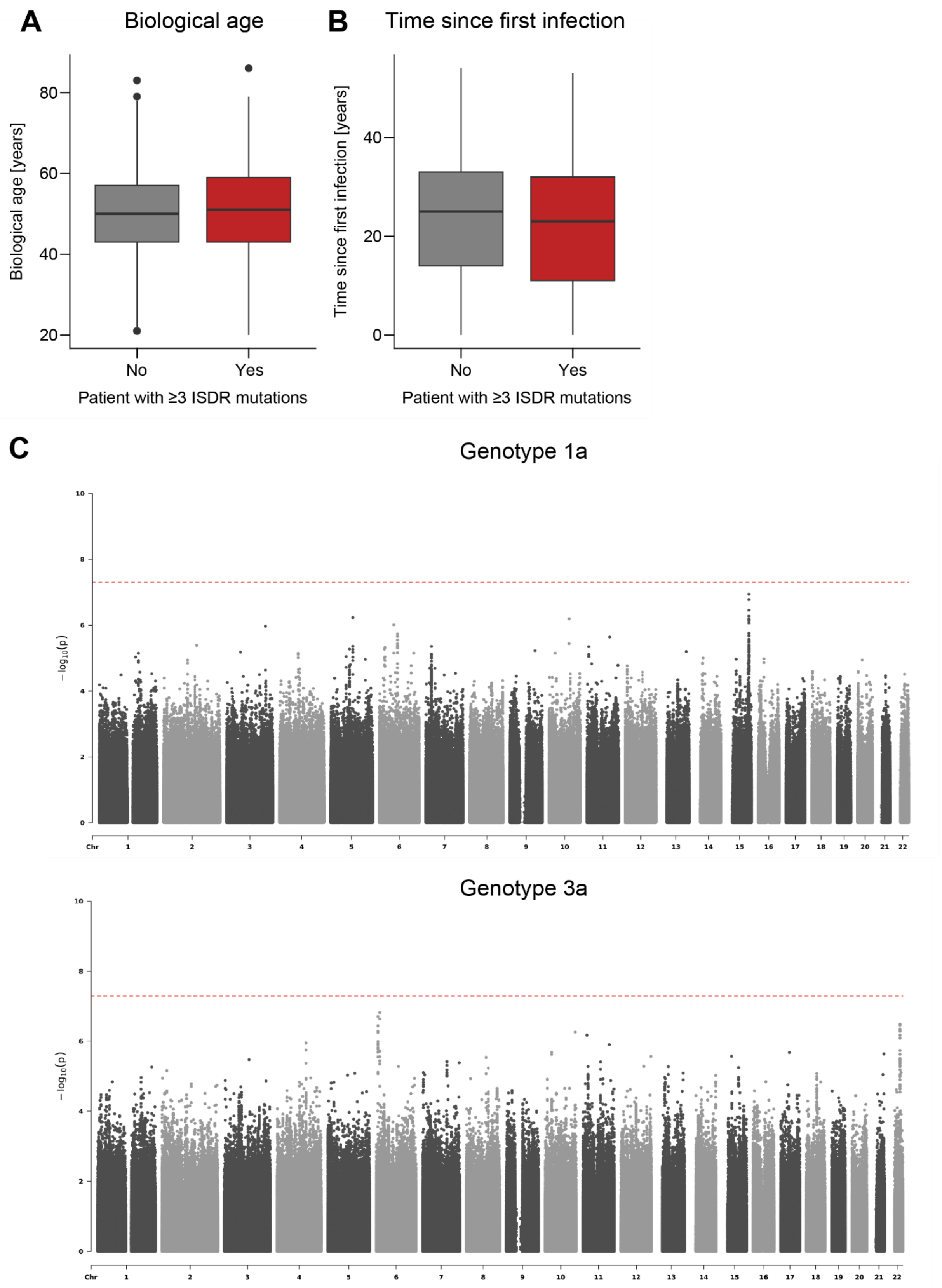

Fig. S12: Genetic and temporal determinants of high RF. A) Fraction of high replicators in the context of biological age. 273 potential high replicators were compared to 1841 potential low replicators. B) Fraction of high replicators in the context of time since first infection. 130 potential high replicators were compared to 843 potential low replicators. A&B) Data based on the HCV

Research UK cohort[8]. Statistical significance was determined using a two-sided Student's t-test. In none of the cases, a significant enrichment of potential high replicators was detected. C) Association between SNPs and patients being infected by a potential high replicator. 1347 gt1a and 1181 gt3a patients were analysed. The dashed red line indicates the threshold for statistical significance determined via logistic regression.

Table S1: Oligo nucleotides used and generated during this project.

Name	Sequence (5‘-3‘)	Description	Gt
A_9416	CAGGATGGCCTATTGGCCTGGAG	cDNA generation[9]	1b
S_59	TGTCTTCACGCAGAAAGCGTCTAG	Nested PCR from cDNA to amplify N-terminal half of HCV genome[9]	1b
S_93	AAGCGTCTAGCCATGGCGTT		1b
A_5982	TGACCAGGTCCTCGGTGGAG		1b
A_6103	GCTATCAGCCGGTTCATCCACTGC		1b
S_4540	GTAACGAGCTCGCCGCGCAGCTGTC	Nested PCR from cDNA to amplify C-terminal half of HCV genome[9]	1b
S_5120	GTGCGCCAGGGCTCAGGCTCCACC		1b
A_9364	GGAGCAGGTAGATGCCTACCCCTAC		1b
A_9386	TTAGCTCCCCGTTCATCGGTTGG		1b
S_6767	ATTCCAGGTCGGGCTCAA	Nested PCR from cDNA to amplify ReED	1b
S_6843	ACTTCCATGCTCACCGACCC		1b
A_7501	AGAGGGGGCATGGAGGAGTA		1b
A_7552	ACGGTAGACCAAGACCCGTC		1b
A9400_1a	GGCCGGAGTGTTTACCCCAACCTTC	cDNA generation[10]	1a
S6583_1a	GCGCTGTGGAGGGTGTCTGC	Nested PCR from cDNA to amplify ReED	1a
S6853_1a	TGACGTCCATGCTCACTG		1a
A7416_1a	CGGAAGTTGAGGAGCTGCC		1a
A7538_1a	AGATCCGGATCCCCAGGCTCC		1a
A9429_3a	ATGGAGTGTTATCCTACCAGC	cDNA generation	3a
S6872_3a	TGACCTCGATGTTGAGAGACC	Nested PCR from cDNA to amplify ReED	3a
S6826_3a	ATAGGATCTCAACTCCCCTGTG		3a
A7488_3a	CTTGGAAGTAGTGCTGGACTG		3a
A7622_3a	ACGCTCTGCTCCTCGCTGTC		3a

Table S2: Patient characteristics of the gt1 post LTX patients. na = not available.

Name	Source	Date sequenced pre LTX sample	LTX Date	Date sequenced post LTX sample	Date biopsy post LTX	Accession No.	Note
FCH1	Barcelona	17.03.11	17.03.11	02.06.11	02.05.11	MK092096/7	
FCH2	Barcelona	21.02.12	21.02.12	19.04.12	na	MK092098/9	
FCH3	Barcelona	09.10.12	09.10.12	12.11.12	na	MK092100/1	
FCH4	Barcelona	22.11.13	22.11.13	18.02.14	12.02.14	MK092102/3	
FCH5	Barcelona	01.01.14	01.01.14	31.03.14	06.02.14	MK092104/5	

FCH6	Barcelona	04.12.09	04.12.09	26.02.10	na	PV083188/9	
FCH7	Barcelona	22.02.12	22.02.12	11.06.12	na	PV083190/1	
FCH8	Barcelona	13.03.12	13.03.12	13.06.12	na	PV083192/3	
FCH9	Barcelona	25.03.12	25.03.12	14.05.12	na	PV083194/5	
FCH10	Barcelona	30.04.13	30.04.13	05.08.13	na	PV083196/7	
FCH11	Barcelona	24.07.13	24.07.13	29.08.13	na	PV083198	
FCH12	Barcelona	10.10.13	10.10.13	29.01.14	09.12.13	PV083199	
1a_FCH1	Barcelona	na	16.01.13	07.02.13	na	PX390139	
Non-FCH1	Barcelona	05.01.01	05.01.01	29.03.01	na	MK092106/7	
Non-FCH2	Barcelona	13.04.07	13.04.07	20.06.07	na	MK092108/9	
Non-FCH3	Barcelona	09.09.01	09.09.01	16.11.01	na	MK092110/1	
Non-FCH4	Heidelberg	na	12.11.09	10.01.12	na	PV083201	Previous LTX on 09.08.07
Non-FCH5	Heidelberg	na	29.03.12	24.08.12	na	PV083202	
Non-FCH6	Heidelberg	na	08.10.11	13.11.12	na	PV083203	Previous LTX on 28.09.11
Non-FCH7	Freiburg	na	12.2011	07.11.12	na	PV083204	
Non-FCH8	Essen	na	03.12.12	03.06.13	na	PV083205	
Non-FCH9	Essen	na	05.12.12	05.06.13	na	PV083206	
LTX_DAA1	Essen	na	17.02.14	28.04.14	na	PV083200	RBV/DCV/ SOF treatment start 20.05.14

Table S3: DNA plasmids used and generated during this project.

Name	Description
**pFK**	Plasmid containing a T7 promotor for *in vitro* transcription
pFK i341 NS3-3' Con1	Bicistronic SGR of the prototype gt1b wildtype isolate[11]
pFK i341 NS3-3' Con1 ΔGDD	Replication deficient negative control
pFK i341 NS3-3' GLT1	gt1b wildtype isolate[9]
pFK JC1	Prototype isolate for infection experiments[1]
pFK JcN2A	JC1 with a Nluc reporter[12]
pFK JcN2A ΔGDD	Replication deficient negative control
pFK JcN2A ΔE1E2	Infection deficient negative control
pFK i389 NS3-3' GLT1	Monocistronic GLT1 SGR
pFK i389 NS3-3' preLTX1α	Monocistronic SGRs of the different timepoints of the GLT1 patient
pFK i389 NS3-3' preLTX1β	
pFK i389 NS3-3' postLTX1	

pFK i389 NS3-3' 6 w postLTX2	
pFK i389 preLTX1α G5A.ReED	Chimeras of the pre LTX timepoints harbouring the GLT1.ReED or ISDR
pFK i389 preLTX1β G5A.ISDR	
pFK i389 preLTX1β G5A.ReED	
pFK i389 N2A preLTX1α/C3JFH1	Chimeras of the indicated assembly module and the JFH1 replicase to assess particle production
pFK i389 N2A preLTX1β/C3JFH1	
pFK i389 N2A preLTX1γ/C3JFH1	
pFK i389 N2A postLTX1/C3JFH1	
pFK i341 NS3-3' Con1 gt1b.ReED	Con1 based chimera harbouring the gt1b consensus ReED
pFK i341 NS3-3' Con1 FCH1.ReED	Con1 based chimeras harbouring patient derived ReEDs from time points pre or post LTX
pFK i341 NS3-3' Con1 FCH2_pre.ReED	
pFK i341 NS3-3' Con1 FCH2_post.ReED	
pFK i341 NS3-3' Con1 FCH3.ReED	
pFK i341 NS3-3' Con1 FCH4_pre.ReED	
pFK i341 NS3-3' Con1 FCH4_post.ReED	
pFK i341 NS3-3' Con1 FCH5_pre.ReED	
pFK i341 NS3-3' Con1 FCH5_post.ReED	
pFK i341 NS3-3' Con1 BHCV1.ReED	
pFK i341 NS3-3' Con1 Non-FCH1.ReED	
pFK i341 NS3-3' Con1 Non-FCH2.ReED	
pFK i341 NS3-3' Con1 Non-FCH3.ReED	
pFK i341 NS3-3' Con1 FCH6_pre.ReED	
pFK i341 NS3-3' Con1 FCH6_post.ReED	
pFK i341 NS3-3' Con1 FCH7_pre.ReED	
pFK i341 NS3-3' Con1 FCH7_post.ReED	
pFK i341 NS3-3' Con1 FCH8_pre.ReED	
pFK i341 NS3-3' Con1 FCH8_post.ReED	
pFK i341 NS3-3' Con1 FCH9_pre.ReED	
pFK i341 NS3-3' Con1 FCH9_post.ReED	
pFK i341 NS3-3' Con1 FCH10_pre.ReED	
pFK i341 NS3-3' Con1 FCH10_post.ReED	
pFK i341 NS3-3' Con1 FCH11.ReED	
pFK i341 NS3-3' Con1 FCH12.ReED	
pFK i341 NS3-3' Con1 Non-FCH4.ReED	
pFK i341 NS3-3' Con1 Non-FCH5.ReED	
pFK i341 NS3-3' Con1 Non-FCH6.ReED	
pFK i341 NS3-3' Con1 Non-FCH7.ReED	
pFK i341 NS3-3' Con1 Non-FCH8.ReED	
pFK i341 NS3-3' Con1 Non-FCH9.ReED	
pFK i341 NS3-3' Con1 EC1.ReED	
pFK i341 NS3-3' GLT1 Con1.ReED	GLT1 based chimera harbouring the Con1 ReED
pFK N2A Con1 fl. ΔGDD	Replication deficient negative control
pFK N2A Con1 fl.	Con1 WT or Con1 based chimeric replicons to assess replication of full-length constructs
pFK N2A Con1 fl. GLT1.ReED	
pFK N2A Con1 fl. FCH3.ReED	
pFK i341 NS3-3' FCH3	SGR of the consensus sequence of patient FCH3
pFK i341 NS3-3' FCH4	SGR of the consensus sequence of patient FCH4

pFK i341 NS3-3' H77	Prototype gt1a isolate[13]
pFK i341 NS3-3' H77 1a_LTX1.ReED	H77 based chimeras harbouring patient derived ReEDs
pFK i341 NS3-3' H77 1a_LTX2.ReED	
pFK i341 NS3-3' H77 1a_LTX3.ReED	
pFK i341 NS3-3' H77 1a_LTX4.ReED	
pFK i341 NS3-3' H77 1a_LTX5.ReED	
pFK i341 NS3-3' H77 1a_LTX6.ReED	
pFK i341 NS3-3' H77 1a_LTX7.ReED	
pFK i341 NS3-3' H77 1a_LTX8.ReED	
pFK i341 NS3-3' H77 1a_LTX9.ReED	
pFK i341 NS3-3' H77 1a_FCH1.ReED	
pFK i341 NS3-3' H77 1a_FCH2.ReED	
pFK i341 NS3-3' H77 Con1.ReED	
pFK i341 NS3-3' H77 GLT1.ReED	
pFK i341 NS3-3' H77 gt1a_cons.ReED	H77 based chimera harbouring the gt1a consensus ReED
pFK i341 NS3-3' H77 gt1a_cons.ReED P2209L	H77 gt1a_cons.ReED based ISDR mutants
pFK i341 NS3-3' H77 gt1a_cons.ReED A2217T	
pFK i341 NS3-3' H77 gt1a_cons.ReED D2220N	
pFK i341 NS3-3' H77 gt1a_cons.ReED I2227V	
pFK i341 NS3-3' H77 gt1a_cons.ReED N2240D	
pFK i341 NS3-3' Con1 GLT1.NS34A	Chimeras for mappings between Con1 and GLT1
pFK i341 NS3-3' Con1 GLT1.NS4B	
pFK i341 NS3-3' Con1 GLT1.NS5A	
pFK i341 NS3-3' Con1 GLT1.NS5B	
pFK i341 NS3-3' Con1 GLT1.AHD1	
pFK i341 NS3-3' Con1 GLT1.LCS1D2	
pFK i341 NS3-3' Con1 GLT1.LCS2D3	
pFK i341 NS3-3' Con1 GLT1.LCS1	
pFK i341 NS3-3' Con1 GLT1.D2	
pFK i341 NS3-3' Con1 GLT1.ISDR	
pFK i341 NS3-3' Con1 G.2209-2280	
pFK i341 NS3-3' Con1 G.2209-2283	
pFK i341 NS3-3' Con1 G.2209-2285	
pFK i341 NS3-3' Con1 G.2209-2303	
pFK i341 NS3-3' Con1 gt1b.C-term FCH2.ISDR	Con1 based chimera harboring the gt1b consensus C-term and patient derived ISDRs
pFK i341 NS3-3' Con1 gt1b.C-term FCH3.ISDR	
pFK i341 NS3-3' Con1 gt1b.ReED FCH5_Insert	Con1 based chimera harboring the gt1b consensus ReED and mutations in the ISDR
pFK i341 NS3-3' Con1 gt1b.ReED FCH11_Insert	
pFK i341 NS3-3' Con1 gt1b.ReED P2209L	
pFK i341 NS3-3' Con1 gt1b.ReED P2209A	
pFK i341 NS3-3' Con1 gt1b.ReED P2209V	
pFK i341 NS3-3' Con1 gt1b.ReED P2209I	
pFK i341 NS3-3' Con1 gt1b.ReED T2217A	
pFK i341 NS3-3' Con1 gt1b.ReED H2218Q	
pFK i341 NS3-3' Con1 gt1b.ReED A2224V	
pFK i341 NS3-3' Con1 gt1b.ReED R2234Q	

pFK i341 NS3-3' Con1 gt1b.ReED N2240D	
pFK i341 NS3-3' Con1 FCH8.ReED A2209P	Con1 based chimera harboring patient derived ReEDs and a point mutation
pFK i341 NS3-3' Con1 FCH10.ReED V2209P	
pFK i341 NS3-3' Con1 FCH12.ReED I2209P	
**pUC**	Plasmid containing a T7 promotor for *in vitro* transcription
pUC i389 NS3-3' BHCV1	SGR of the consensus sequence of patient BHCV1[14]
pUC i387 NS3-3' S52	Prototype gt3a isolate[15]
pUC i387 NS3-3' S52 gt3a_cons.ReED	S52 based chimera harbouring the gt3a consensus ReED
pUC i387 NS3-3' S52 3a_LTX1.ReED	S52 based chimeras harbouring patient derived ReEDs
pUC i387 NS3-3' S52 3a_LTX2.ReED	
pUC i387 NS3-3' S52 3a_LTX3.ReED	
pUC i387 NS3-3' S52 3a_LTX4.ReED	
pUC i387 NS3-3' S52 3a_LTX5.ReED	
pUC i387 NS3-3' S52 3a_LTX6.ReED	
pUC i387 NS3-3' S52 3a_LTX7.ReED	
pUC i387 NS3-3' S52 3a_LTX8.ReED	
pUC i387 NS3-3' S52 3a_LTX9.ReED	
pUC i387 NS3-3' S52 3a_LTX10.ReED	
pUC i387 NS3-3' S52 3a_LTX11.ReED	
pUC i387 NS3-3' S52 3a_LTX12.ReED	
pUC i387 NS3-3' S52 3a_LTX13.ReED	
pUC i387 NS3-3' S52 3a_LTX14.ReED	
pUC i387 NS3-3' S52 3a_LTX15.ReED	
pUC i387 NS3-3' S52 3a_LTX16.ReED	
pUC i387 NS3-3' S52 3a_LTX17.ReED	
pUC i387 NS3-3' S52 gt3a_cons.ReED P2209L	S52 gt3a_cons.ReED based ISDR mutants
pUC i387 NS3-3' S52 gt3a_cons.ReED Q2216H	
pUC i387 NS3-3' S52 gt3a_cons.ReED P2219L	
pUC i387 NS3-3' S52 gt3a_cons.ReED P2220L	
pUC i387 NS3-3' S52 gt3a_cons.ReED H2221Y	
pUC i387 NS3-3' S52 gt3a_cons.ReED N2240T	
pUC i387 NS3-3' ED43	Prototype gt4a isolate[15]
pUC i387 NS3-3' ED43 4a_cons.ReED	ED43 based chimera harbouring the gt4a consensus ReED
pUC i387 NS3-3' ED43 Pat1.ReED	ED43 based chimeras harbouring patient derived ReEDs
pUC i387 NS3-3' ED43 Pat2.ReED	
pUC i387 NS3-3' ED43 Pat3.ReED	
pUC i387 NS3-3' ED43 Pat4.ReED	
pUC i387 NS3-3' ED43 Pat5.ReED	
pUC i387 NS3-3' ED43 Pat6.ReED	
**Other**	
pCMV DR 8.74	Production of HCVpp
pCMV Luc	
pczVSV-Gwt	
pcDNA3cE1E2Con1	
pcDNA3cE1E2 preLTX1α	

pcDNA3cE1E2 preLTX1β	
pcDNA3cE1E2 preLTX1γ	
pcDNA3cE1E2 postLTX1	
pcDNA3cE1E2 GLT1	

References

1. Pietschmann, T., *et al.* Construction and characterization of infectious intragenotypic and intergenotypic hepatitis C virus chimeras. *Proc. Natl. Acad. Sci. U. S. A* **103**, 7408-7413 (2006).
2. Enomoto, N., *et al.* Comparison of full-length sequences of interferon-sensitive and resistant hepatitis C virus 1b. Sensitivity to interferon is conferred by amino acid substitutions in the NS5A region. *The Journal of Clinical Investigation* **96**, 224-230 (1995).
3. Gale, M., Jr., *et al.* Control of PKR protein kinase by hepatitis C virus nonstructural 5A protein: molecular mechanisms of kinase regulation. *Mol Cell Biol* **18**, 5208-5218 (1998).
4. Dujardin, M., *et al.* Cyclophilin A allows the allosteric regulation of a structural motif in the disordered domain 2 of NS5A and thereby fine-tunes HCV RNA replication. *Journal of Biological Chemistry* **294**, 13171-13185 (2019).
5. Pickett, B.E., *et al.* ViPR: an open bioinformatics database and analysis resource for virology research. *Nucleic Acids Res* **40**, D593-598 (2012).
6. Gambato, M., *et al.* Hepatitis C virus intrinsic molecular determinants may contribute to the development of cholestatic hepatitis after liver transplantation. *Journal of General Virology* **100**, 63-68 (2019).
7. Coss, S.L., *et al.* CD4+ T cell restoration and control of hepatitis C virus replication after childbirth. *The Journal of Clinical Investigation* **130**, 748-753 (2020).
8. McLauchlan, J., *et al.* Cohort Profile: The Hepatitis C Virus (HCV) Research UK Clinical Database and Biobank. *Int J Epidemiol* **46**, 1391-1391h (2017).
9. Heuss, C., *et al.* A Hepatitis C virus genotype 1b post-transplant isolate with high replication efficiency in cell culture and its adaptation to infectious virus production in vitro and in vivo. *PLOS Pathogens* **18**, e1010472 (2022).
10. Esser-Nobis, K., *et al.* Analysis of hepatitis C virus resistance to silibinin in vitro and in vivo points to a novel mechanism involving nonstructural protein 4B. *Hepatology* **57**, 953-963 (2013).
11. Lohmann, V., *et al.* Replication of subgenomic hepatitis C virus RNAs in a hepatoma cell line. *Science* **285**, 110-113 (1999).
12. Schult, P., *et al.* microRNA-122 amplifies hepatitis C virus translation by shaping the structure of the internal ribosomal entry site. *Nature Communications* **9**, 2613 (2018).
13. Blight, K.J., McKeating, J.A., Marcotrigiano, J. & Rice, C.M. Efficient replication of hepatitis C virus genotype 1a RNAs in cell culture. *J. Virol* **77**, 3181-3190 (2003).
14. Koutsoudakis, G., *et al.* Cell Culture Replication of a Genotype 1b Hepatitis C Virus Isolate Cloned from a Patient Who Underwent Liver Transplantation. *PloS one* **6**, e23587 (2011).
15. Gottwein, J.M., *et al.* Novel infectious cDNA clones of hepatitis C virus genotype 3a (strain S52) and 4a (strain ED43): genetic analyses and in vivo pathogenesis studies. *J. Virol* **84**, 5277-5293 (2010).
