# Supplementary material for: Highly replicating hepatitis C virus variants emerge in immunosuppressed patients causing severe disease
Source: Nat Commun. 2025 Dec 7;16:10948. doi: 10.1038/s41467-025-67174-w (PMC12686413; doi:10.1038/s41467-025-67174-w)
Supplement: Supplementary file 3 — Reporting Summary [file 41467_2025_67174_MOESM3_ESM.pdf]

nature portfolio

Corresponding author(s): Prof. Dr. Volker Lohmann

Last updated by author(s): Oct 30, 2025

Reporting Summary

Nature Portfolio wishes to improve the reproducibility of the work that we publish. This form provides structure for consistency and transparency in reporting. For further information on Nature Portfolio policies, see our Editorial Policies and the Editorial Policy Checklist.

Statistics

For all statistical analyses, confirm that the following items are present in the figure legend, table legend, main text, or Methods section.

n/a	Confirmed	
☐	☒	The exact sample size (*n*) for each experimental group/condition, given as a discrete number and unit of measurement
☐	☒	A statement on whether measurements were taken from distinct samples or whether the same sample was measured repeatedly
☐	☒	The statistical test(s) used AND whether they are one- or two-sided<br>*Only common tests should be described solely by name; describe more complex techniques in the Methods section.*
☒	☐	A description of all covariates tested
☒	☐	A description of any assumptions or corrections, such as tests of normality and adjustment for multiple comparisons
☐	☒	A full description of the statistical parameters including central tendency (e.g. means) or other basic estimates (e.g. regression coefficient) AND variation (e.g. standard deviation) or associated estimates of uncertainty (e.g. confidence intervals)
☐	☒	For null hypothesis testing, the test statistic (e.g. *F*, *t*, *r*) with confidence intervals, effect sizes, degrees of freedom and *P* value noted<br>*Give P values as exact values whenever suitable.*
☒	☐	For Bayesian analysis, information on the choice of priors and Markov chain Monte Carlo settings
☒	☐	For hierarchical and complex designs, identification of the appropriate level for tests and full reporting of outcomes
☒	☐	Estimates of effect sizes (e.g. Cohen's *d*, Pearson's *r*), indicating how they were calculated

Our web collection on statistics for biologists contains articles on many of the points above.

Software and code

Policy information about availability of computer code

Data collection	No software was used for data collection
Data analysis	NGS data were analysed using Burrows-Wheeler aligner (v. 0.7.17), the genome analysis toolkit (v. 4.1.4.1) and bcftools (v. 1.22). Phylogenetic trees were generated with the tools of the virus and pathogen database and analysis resource (VipR) and visualised using FigTree (v. 1.4.4). Sequence alignments were performed with ClustalOmega visualised with Jalview (v. 2.11.4) and analysed with R (v. 4.5.1). IHC images were visualised using Aperio ImageScope (Leica Biosystems, v. 12.3). Statistical analyses were performed using GraphPad Prism (Graphpad Software, v. 8.4.3) or R (v. 4.5.1).

For manuscripts utilizing custom algorithms or software that are central to the research but not yet described in published literature, software must be made available to editors and reviewers. We strongly encourage code deposition in a community repository (e.g. GitHub). See the Nature Portfolio guidelines for submitting code & software for further information.

Data

Policy information about availability of data

All manuscripts must include a data availability statement. This statement should provide the following information, where applicable:

- Accession codes, unique identifiers, or web links for publicly available datasets
- A description of any restrictions on data availability
- For clinical datasets or third party data, please ensure that the statement adheres to our policy

Parts of the FCH cohort were previously published under the accession numbers MK092096- MK092105 (FCH1-5) [https://www.ncbi.nlm.nih.gov/nuccore/?term=MK092096%3AMK092105+%5Bpacc%5D], MK092106-MK092111 (Non-FCH1-3) [https://www.ncbi.nlm.nih.gov/nuccore/?term=MK092106%3AMK092111++%5Bpacc%5D], OM222702 (GLT1) [https://www.ncbi.nlm.nih.gov/nuccore/OM222702], HQ719473 (BHCV1) [https://www.ncbi.nlm.nih.gov/nuccore/HQ719473] and JQ914274 (1a_FCH2) [https://www.ncbi.nlm.nih.gov/nuccore/JQ914274]. Sequence information on all other FCH patients generated during this study is deposited in GenBank under the following accessions: PV083181-PV083206 [https://www.ncbi.nlm.nih.gov/nuccore/?term=PV083181%3APV083206+%5Bpacc%5D], PX390011-PX390139 [https://www.ncbi.nlm.nih.gov/nuccore/?term=PX390011%3APX390139+%5Bpacc%5D]. NGS data from the GLT1 patient are deposited in the sequence read archive (SRA) under the BioProject accession PRJNA1214216 [https://www.ncbi.nlm.nih.gov/bioproject/?term=PRJNA1214216]. All other data generated during this study are included in this published article (and its supplementary information files). Source data are provided with this paper.

Research involving human participants, their data, or biological material

Policy information about studies with human participants or human data. See also policy information about sex, gender (identity/presentation), and sexual orientation and race, ethnicity and racism.

Reporting on sex and gender	not applicable
Reporting on race, ethnicity, or other socially relevant groupings	not applicable
Population characteristics	not applicable
Recruitment	not applicable
Ethics oversight	This study was approved by the ethics committee of the medical faculty of Heidelberg University (ethics votes: S-399/2012, S-720/2022 and S-743/2023). 12 liver samples were provided by the tissue bank of the German Center for Infection Research (DZIF, Heidelberg, Germany) in accordance with the regulations of the tissue bank and the approval of the ethics committee of Heidelberg University (ethics vote: S-399/2012). One liver sample of an HCV negative patient suffering from cholestatic liver disease was approved by the ethics committee of Klinikum rechts der Isar (MRI, Munich) (ethics vote: 518/19 S). The Montreal Hepatitis C Cohort study (HEPCO) is approved by the Research Ethics Committee of the Centre de Recherche du Centre Hospitalier de l'Université de Montréal (CRCHUM) (Approval number: SL 05.014). The studies were conducted in accordance with the local legislation and institutional requirements. The participants provided their written informed consent to participate in this study.

Note that full information on the approval of the study protocol must also be provided in the manuscript.

Field-specific reporting

Please select the one below that is the best fit for your research. If you are not sure, read the appropriate sections before making your selection.

☒ Life sciences ☐ Behavioural & social sciences ☐ Ecological, evolutionary & environmental sciences

For a reference copy of the document with all sections, see nature.com/documents/nr-reporting-summary-flat.pdf

Life sciences study design

All studies must disclose on these points even when the disclosure is negative.

Sample size	We performed at least n=3 independent biological replicates for all experiments. This adheres to common standards of good scientific practice. For patient cohorts, all available data was used to maximize the sample size and thus the robustness of the analysis
Data exclusions	No data were excluded from the analysis
Replication	All attempts at replication were successful
Randomization	This is not relevant to our study since all experiments are built on distinct constructs and mutants.
Blinding	Experiments are built on distinct constructs and mutants. Therefore, blinding is not relevant or possible in this setting.

Reporting for specific materials, systems and methods

We require information from authors about some types of materials, experimental systems and methods used in many studies. Here, indicate whether each material, system or method listed is relevant to your study. If you are not sure if a list item applies to your research, read the appropriate section before selecting a response.

Materials & experimental systems

n/a	Involved in the study
☐	☒ Antibodies
☐	☒ Eukaryotic cell lines
☒	☐ Palaeontology and archaeology
☒	☐ Animals and other organisms
☒	☐ Clinical data
☒	☐ Dual use research of concern
☒	☐ Plants

Methods

n/a	Involved in the study
☒	☐ ChIP-seq
☒	☐ Flow cytometry
☒	☐ MRI-based neuroimaging

Antibodies

Antibodies used	HCV NS5A Mouse monoclonal “9E10”, IgG2; Lindenbach et al., 2005; diluted 1:4,000
Validation	Immunohistochemistry with HCV negative liver samples (Reiss et al., 2011; Figure 5&S3, Table S4)

Eukaryotic cell lines

Policy information about cell lines and Sex and Gender in Research

Cell line source(s)	Friebe, P., Boudet, J., Simorre, J. P. & Bartenschlager, R. Kissing-loop interaction in the 3' end of the hepatitis C virus genome essential for RNA replication. J Virol 79, 380-392 (2005). https://doi.org/10.1128/JVI.79.1.380-392.2005<br>Koutsoudakis, G., Herrmann, E., Kallis, S., Bartenschlager, R. & Pietschmann, T. The level of CD81 cell surface expression is a key determinant for productive entry of hepatitis C virus into host cells. J Virol 81, 588-598 (2007). https://doi.org/10.1128/JVI.01534-06<br>Heuss, C., et al. A Hepatitis C virus genotype 1b post-transplant isolate with high replication efficiency in cell culture and its adaptation to infectious virus production in vitro and in vivo. PLOS Pathogens 18, e1010472 (2022).
Authentication	Multiplex human cell line authentication test
Mycoplasma contamination	negative
Commonly misidentified lines (See ICLAC register)	no commonly misidentified cell lines have been used in this study

Plants

Seed stocks	*Report on the source of all seed stocks or other plant material used. If applicable, state the seed stock centre and catalogue number. If plant specimens were collected from the field, describe the collection location, date and sampling procedures.*
Novel plant genotypes	*Describe the methods by which all novel plant genotypes were produced. This includes those generated by transgenic approaches, gene editing, chemical/radiation-based mutagenesis and hybridization. For transgenic lines, describe the transformation method, the number of independent lines analyzed and the generation upon which experiments were performed. For gene-edited lines, describe the editor used, the endogenous sequence targeted for editing, the targeting guide RNA sequence (if applicable) and how the editor was applied.*
Authentication	*Describe any authentication procedures for each seed stock used or novel genotype generated. Describe any experiments used to assess the effect of a mutation and, where applicable, how potential secondary effects (e.g. second site T-DNA insertions, mosiacism, off-target gene editing) were examined.*
